# Harvested reservoir computing from road traffic dynamics

**DOI:** 10.1038/s41598-025-30016-2

**Published:** 2025-11-27

**Authors:** Ryunosuke Fukuzaki, Takahiro Noguchi, Hiroyasu Ando

**Affiliations:** 1 Graduate School of Science and Technology, University of Tsukuba, Tsukuba, 305-8573 Japan; 2 Faculty of Economics, Keio University, Tokyo, 108-0073 Japan; 3 WPI-Advanced Institute for Materials Research (WPI-AIMR), Tohoku University, Sendai, 980-8577 Japan

**Keywords:** Reservoir computing, Road traffic, Computation Harvesting, Engineering, Mathematics and computing, Physics

## Abstract

Reservoir computing (RC) has gained attention as an efficient machine learning method for time series prediction because of its low computational costs and simple learning process. Herein, we propose the Harvested Reservoir Computing (HRC) framework which treats complex real-world dynamics as spontaneously emerging physical reservoirs. As an instance of HRC, we introduce Road Traffic Reservoir Computing (RTRC), whereby dynamical traffic flow patterns are harnessed as natural computational resources to predict future traffic states in experiments. Unlike conventional reservoir computing, this approach requires no explicit reservoir design, but instead “harvests” the intrinsic dynamics of traffic as a physical reservoir. Experiments using a scaled traffic model and numerical simulations on a grid road network demonstrate that the framework’s prediction accuracy is highly dependent on traffic density. An optimal density range is identified within which prediction performance is maximized because of a tradeoff between nonlinearity and short-term memory. These findings highlight the potential of complex real-world dynamics as viable components within computational frameworks.

## Introduction

In recent years, advances in machine learning, big data, and artificial intelligence (AI) have accelerated rapidly. Particularly, deep learning has achieved great success in various fields of information processing, including image processing, speech recognition, and natural language processing. However, deep learning typically requires enormous high-performance computational resources and extensive datasets for training, leading to surging computational demand as these technologies become more widespread. In this context, reservoir computing (RC)^1–5^, a machine-learning technique that enables fast and low-cost learning, has garnered increasing attention, especially in the field of time series information processing^6–8^. Furthermore, in recent years, RC implementation and application have been demonstrated in numerous fields such as biomedical science^9–11^, chemistry^12,13^, manufacturing processes^14,15^, security^16,17^, natural phenomena^18,19^, and finance^20–22^. Unlike training in conventional artificial neural networks, a recurrent neural network called an Echo State Network (ESN)^1,3^, which is an exemplification of RC, does not adjust all connection weights of networks. Instead, only the readout weights are adjusted. Those weights can be obtained easily through linear regression. This characteristic makes RC an attractive technique for low-cost learning. Furthermore, because of the RC design, physical reservoir computing (PRC), which replaces the recurrent neural network with a nonlinear physical system, has become an active research area^23,24^. According to the characteristics of PRC, the implementation of reservoirs in hardware is expected to engender the development of computational systems with considerably lower energy consumption.

As a direction for additional advancement in this field, recent studies have introduced information processing techniques that use complex real-world dynamics directly as part of reservoir computing^25–27^. These approaches extend beyond conventional PRC by exploiting natural complexity itself for reservoir computing. Traffic flow is a representative example. Although artificially constructed, it exhibits universal characteristics resembling those of natural phenomena. Traffic flow can be interpreted as a physical reservoir for predicting future states^28–30^. We designate this methodology as *Road Traffic Reservoir Computing (RTRC)*. Unlike conventional RC, for which the reservoir is deliberately designed, RTRC relies on naturally arising traffic dynamics, which vary with travel demand and which are inherently non-stationary^31–33^. If these dynamics exhibit suitable properties such as nonlinearity and short-term memory, then their computational capability can be expected to emerge or diminish depending on traffic conditions. This expectation suggests that certain real-world systems might function as spontaneously arising reservoirs.

Based on these studies of RTRC, we recently proposed the *Computation Harvesting (CH)*^29,34^ framework, which is designed to “harvest” undesigned dynamics from the real world and to repurpose them as computational resources. Our group has demonstrated CH empirically in both urban traffic and plant motion, demonstrating that physical and social phenomena can function effectively as computational resources without excessive design. Specifically, we examine the framework of RC with the example of CH: we position *Harvested Reservoir Computing (HRC)* as a practical model of CH.

It is evident that the nature of the dynamics harvested as computational resources in HRC depends on the observed environment. Although the effectiveness of RTRC has been demonstrated in previous studies, the specific conditions under which traffic dynamics function as computational resources have not yet been clarified. In other words, it remains unclear under what traffic states computational capability emerges – a crucial theoretical issue for RTRC. For this study, we specifically examine traffic flow as a harvested reservoir and evaluate the performance of RTRC under varying density conditions. Through experimentation with a scaled traffic model and simulations of a simple car-following model^35^ on a grid network, we demonstrate that computational performance depends on traffic density. Then we clarify the conditions under which traffic dynamics function effectively as harvested reservoir computing.

## Related work

This section presents a review of the RC and PRC foundations, followed by a review of recent studies that have used real-world complexity as physical reservoirs. The emphasis of this work is on RTRC as the main application.

### Fundamentals of reservoir computing

**Fig. 1:**
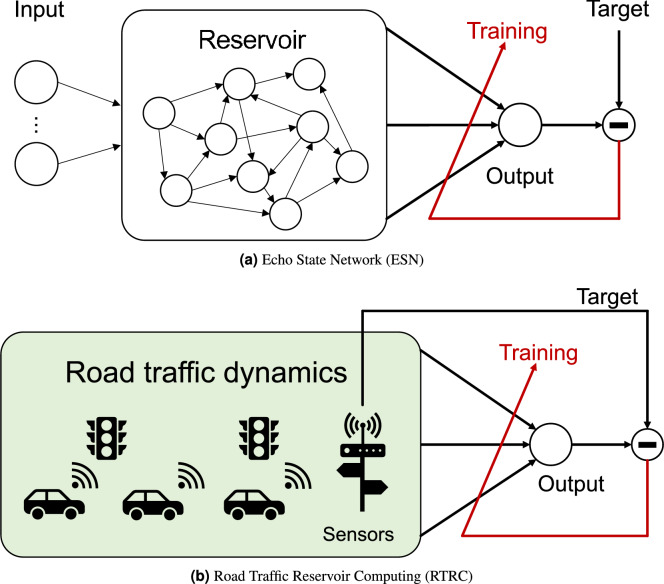
Conceptual illustrations of reservoir computing (RC) (top) and Road Traffic Reservoir Computing (RTRC) (bottom). In standard RC, an artificial dynamical system processes inputs. By contrast, in RTRC, natural traffic flow dynamics are harvested as computational substrates.

The RC machine-learning framework generalizes a specialized model of recurrent neural networks (RNNs). The two key components of RC are the reservoir and the readout. The reservoir is a fixed nonlinear dynamical system that maps input data into a high-dimensional space. The readout, which corresponds to the output layer weights in a neural network, is a linear mapping (matrix). In RC, the input and reservoir connection weights are assigned randomly. They remain fixed. Only the readout weights are adjusted. This approach enables efficient and low-cost learning while maintaining the predictive performance. The following describes the Echo State Network (ESN)^1^: a representative model of RC (Fig. 1a ). The typical update equation of the reservoir $x(t)\in \mathbb {R}^{N_x}$ in ESN is the following, where $N_x$ denotes the number of reservoir sizes as

1 $$\begin{aligned} x(t) = f( W^{\text {in}}u(t) + Wx(t-1) ) , \end{aligned}$$

where $W^{\text {in}}\in \mathbb {R}^{N_x \times N_u}$ and $W \in \mathbb {R}^{N_x \times N_x}$ respectively represent the input weight matrix and the reservoir connection weight matrix. Function *f* is typically a nonlinear function: often the $\tanh$ function. The RC output $\hat{y}(t+1)$ is obtained using a linear combination in the readout layer as

2 $$\begin{aligned} \hat{y}(t+1) = W^{\text {out}} [1; x(t)], \end{aligned}$$

where $W^{\text {out}} \in \mathbb {R}^{(N_x+1)}$ denotes the readout weight matrix. Generally, in matrix notation, it is expressed as $\hat{Y} = {W}^{\text {out}} X$. Here, $\hat{Y} \in \mathbb {R}^{N_y \times T}$ consists of all $\hat{y}$ in the time direction $t = 1, \dots , T$. Similarly, $X \in \mathbb {R}^{(1+N_x) \times T}$ consists of all *x* stacked in the time direction. For simplicity in notation, *X* includes the bias term, i.e., it represents [1; *X*].

The readout weights $W^{\text {out}}$ are calculated using Ridge regression, which minimizes the squared error between the predicted output $\hat{y}(t)$ and the target time series *y*(*t*). The value of $W^{\text {out}}$ is

3 $$\begin{aligned} W^{\text {out}} = Y X^T (XX^T + \beta I)^{-1}, \end{aligned}$$

where $\beta$ is a regularization coefficient and where *I* is the identity matrix. Unlike conventional neural networks that optimize all connection weights via gradient descent, RC emphasizes extraction of the desired information from the reservoir by readout. This characteristic has been extended to PRC, where the role of the reservoir is replaced by physical systems. Various physical systems have been implemented as physical reservoirs, including electronic and optical systems^36–39^, soft robots^40,41^, spin waves^42–45^, and quantum systems^46–49^. The hardware implementation is expected to improve energy efficiency and computational speed, consequently demonstrating that the realization of AI technology is not limited to software: it can also be achieved through the integration of hardware and software.

### Exploiting real-world dynamics as a reservoir

Because they have fewer tunable parameters, RC models are simpler to design than conventional RNNs. However, parameters such as input scaling, the spectral radius of *W*, and reservoir size must still be set carefully. Common ESN variants, including those with leaky integrators^50^ and feedback connections^51^, also require tuning of leakage and feedback rates depending on the task^52^. Generally speaking, most machine learning models rely on hyperparameter optimization to achieve good performance. However, if rich observational data are available, then is such detailed model design always necessary? For complex real-world dynamics, it might be possible to produce accurate predictions using simple readout models alone.

Recent advances in sensing and IoT technologies have made it increasingly feasible to measure dynamic patterns from the real world. Several studies have demonstrated causal inference and predictive capability from undesigned dynamics, such as those of ecological networks^26^ and plant physiology^27^. These works suggest that natural complexity can be exploited for reservoirs. Chen et al.^25^ argued that artificial reservoirs can be extracted from real-world systems in addition to ESNs. Moreover, they proposed the auto-reservoir neural network (ARNN), which leverages spatial information in complex systems for temporal prediction.

Building on this perspective, we proposed the CH computational paradigm^29,34^, which treats undesigned natural phenomena as “computational resources” by directly sensing and extracting their inherent dynamics. Within the framework of natural computation^53^, real-world processes are regarded as computations. These processes are regarded as a resource-efficient and robust information processing mechanism. As a concrete example, we validated CH using plant oscillations to estimate wind speed and direction in a laboratory setting^34^. Plant dynamics caused by wind were recorded on video. Temporal patterns of feature points were used as reservoir dynamics. A classifier that had been trained only on the readout layer achieved over 99% accuracy without direct sensors, relying solely on video data. Performance remained stable when some feature points were missing, demonstrating robustness and generalization. This study demonstrates CH as distinct from traditional PRC in that it uses complex dynamics found in nature directly, without artificial design.

Thus, in HRC, the reservoir state is not generated by a designed device or a neural mapping; it is the measurement vector, and output is obtained by a linear readout. This contrasts with PRC, which uses a purpose-built physical system, and with ARNN, which uses a fixed random multilayer map *F* and solves conjugate STI equations (Fig.1c in Chen et al.^25^). Our focus is to characterize when the harvested real-world dynamics themselves possess sufficient computation capability, without constructing additional reservoir components.

#### Road traffic reservoir computing

Recent research has also shown rapid progress in data-driven prediction and assignment for road traffic^54^. Graph neural networks (GNNs) estimate link flows from OD demand and network topology, and heterogeneous GNNs with virtual OD links markedly improve link-utilization accuracy and out-of-distribution generalization^55^. Additionally, a recent end-to-end study combines an (adaptive) GCN with LSTM and a YOLO-based sensing pipeline to forecast 5-minute speeds on PeMS, reporting promising accuracy^56^. Despite extensive studies on traffic state prediction using deep and graph neural networks, these approaches require large-scale labeled data and heavy retraining whenever traffic conditions or network structures change. However, in real-world transportation systems, the environment is inherently non-stationary, and online retraining is often infeasible. This gap motivates exploring alternative frameworks that can rapidly adapt or operate without explicit training of internal dynamics. RC provides such a framework, yet most prior RC or PRC studies still rely on artificially designed reservoirs. The possibility of directly harvesting naturally existing dynamics such as traffic flow has not been systematically examined. Therefore, clarifying under what conditions real traffic dynamics can serve as an effective “computational reservoir” addresses a key missing link between machine learning theory and complex physical/social systems.

Earlier studies conducted by the authors have proposed methods that exploit traffic dynamics directly to predict future states of traffic flow in the context of reservoir computing, demonstrating their effectiveness in both simulations and real-world data^28,29^. We refer to this approach as RTRC (Fig. 1b ). In numerical simulations, traffic density and external inputs into the phase of traffic signals were predicted using linear combinations of complex traffic dynamics as a reservoir^28^. In real-world applications, intersection passage data were used as a linear model for RTRC to predict future traffic volumes at target intersections based on flows at other locations^29^. These models partially captured the nonlinear structure of traffic dynamics and achieved predictive accuracy comparable to or exceeding that of autoregressive models. Furthermore, a highway case study demonstrated that linear combinations of detector data enabled highly accurate prediction of congestion onset at bottlenecks^30^, consequently outperforming convolutional neural network (CNN) models.

With RTRC, the traffic measurements themselves are treated as the reservoir, thereby eliminating the need for explicit reservoir design. Although earlier studies have demonstrated the potential of this approach, it remains unclear whether all observed traffic dynamics act as effective reservoirs. For example, accuracy of highway congestion prediction improved^30^ when training used only data gathered immediately before and after congestion, rather than the full dataset^57^. This improved accuracy suggests that the effectiveness of traffic flow as a reservoir depends on the dynamical properties of the traffic flow itself.

Conventional RC studies have identified two key reservoir attributes: nonlinearity and short-term memory. Nonlinearity provides diverse responses, whereas short-term memory retains past information. However, excessive nonlinearity reduces memory capacity^58,59^, posing a tradeoff that has been well established^60^. Therefore, we hypothesize that factors altering traffic flow nonlinearity critically influence the RTRC performance. Regarding traffic engineering, flow is well known to increase with density at low levels but it declines once density exceeds a critical point^61–63^. In free-flow regimes, vehicle interactions are limited and nonlinearity is weak. By contrast, at higher densities, interactions among vehicles strengthen, thereby producing more nonlinear dynamics. Consequently, traffic density acts as a key parameter modulating nonlinearity and short-term memory. For this study, we therefore examine the variation of prediction performance with traffic density, with the objective of identifying the conditions under which RTRC performance can be improved.

## Methods

### Scaled traffic system

**Fig. 2:**
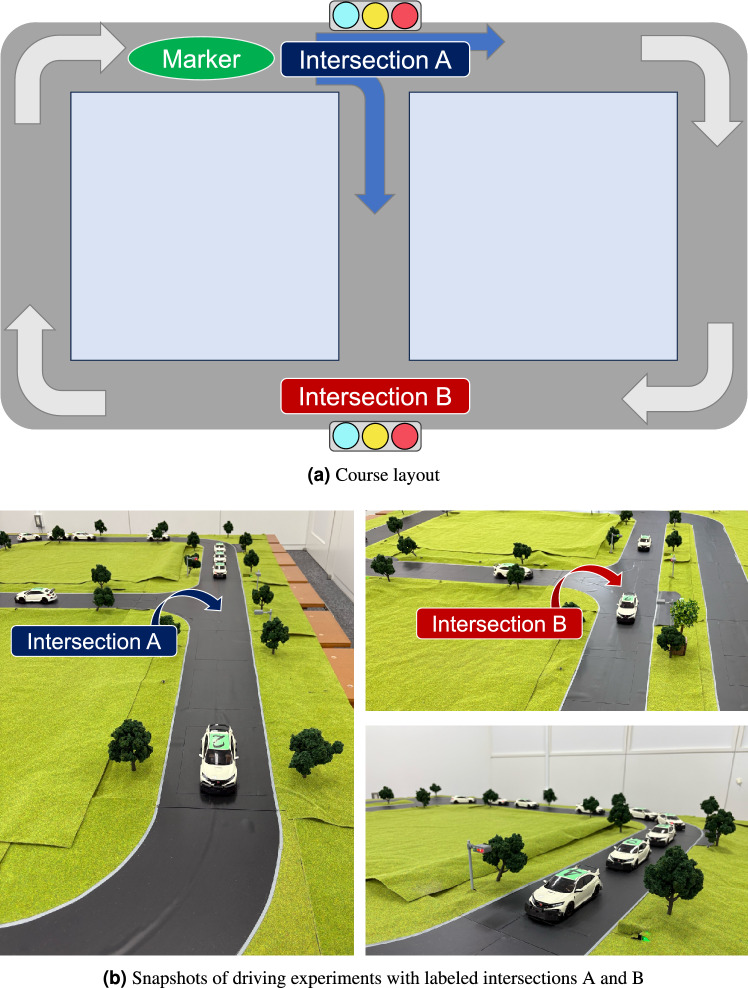
Scaled traffic system. (**a**, top) Schematic layout of the test course, including two signalized intersections labeled as A and B. (**b**, bottom) Photographs of the driving experiments in the scaled traffic system, showing vehicles passing through intersections A (left) and B (right), as well as a vehicle platoon stopped at a signalized intersection (bottom right).

For RTRC to function effectively, appropriate traffic flow characteristics must be inferred. However, modifying real-world traffic properties in experiments presents a challenge. Therefore, we evaluate its computational performance experimentally using traffic reproduced in models and simulations. Hereinafter, we describe the scaled traffic system experiments and computational simulations used to harvest traffic measurement data as the reservoir, together with their respective prediction models.

**Fig. 3:**
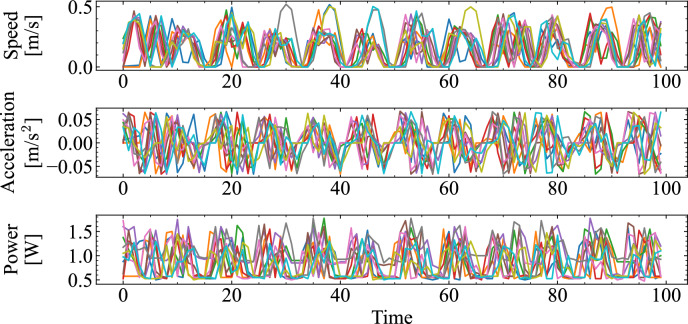
Time series example of vehicle-level data obtained from the scaled traffic system, including speed (top), acceleration (middle), and power consumption (bottom).

The driving experiment was conducted using a scaled traffic system in which 1/27-scale radio-controlled cars navigate a track autonomously to replicate traffic flow^64^. The scaled traffic system serves as a test-bed that is capable of obtaining driving data including various vehicle information, with traffic state data collected each second. Each radio-controlled car is equipped with multiple sensors and wireless devices. Using them, real-time recording of driving data can be achieved as they navigate the diorama, as shown in Fig. 3. Scaled traffic systems with physical vehicles have been used for earlier studies (e.g.,^65–67^) to capture real-world complexities that are difficult to reproduce in simulations. By contrast to the use of full-scale vehicles, radio-controlled cars offer lower costs, enhanced safety, and greater reproducibility by avoiding external factors such as weather, road conditions, and advanced driver-assist features. Furthermore, experiments with scaled traffic systems can be conducted repeatedly in controlled indoor environments.

Figure 2a portrays the layout of the course of the scaled traffic system experiments. For convenience, we refer to the two signalized intersections as Intersection A and Intersection B. At Intersection A, vehicles proceed straight or turn right. The vehicles select their travel routes each time they pass through Intersection A, with equal probability assigned to each choice. Intersection B serves as a merging point for vehicles coming straight from Intersection A and those turning right, thereby preventing collisions. The course has no opposing lanes, but the presence of traffic lights and random route selection leads to variations in vehicle order. The scaled traffic course has no inflow or outflow of vehicles. The number of vehicles corresponds to the traffic density. Consequently, the system density is kept constant. Fig. 2b portrays a radio-controlled car autonomously navigating the course as a self-driving vehicle in the scaled traffic system. Additional details of the vehicles and course design are provided in the Supplementary Information (Section A).

**Fig. 4:**
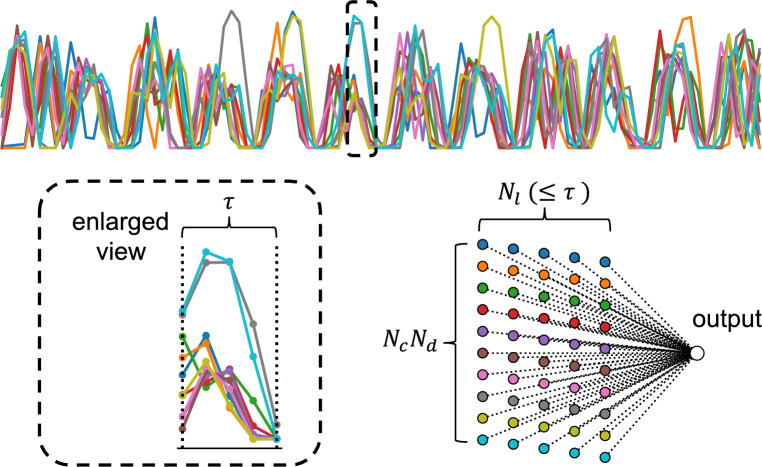
Schematic showing the Road Traffic Reservoir Computing (RTRC) model used for the scaled traffic system. Vehicle data are structured via temporal multiplexing and processed using a linear readout layer for prediction.

The variables measured from this experiment include speed, acceleration, and power consumption. Figure 3 presents an example of driving data obtained from the scaled traffic system. The specific procedure for formatting the driving data as a reservoir state *x* is the following. First, the data of variable $d=1,2,\dots ,N_d$ obtained from each vehicle $c=1,2,\dots ,N_c$ at time step *k* are represented as vehicle measurement data $m_{c,d}(k)$. Furthermore, to enhance the reservoir dimensionality, the time-multiplexing method^36^ is applied, which uses temporally multiplexed data to improve both computational efficiency and performance. Specifically, given a step interval of $\tau$ seconds, each time series is reshaped into a time series sampled at every $\tau$ seconds. As a result, for each step *k*, the measurement $m_{c,d}$, which incorporates a time-lag variable *l*, consists of $N_l$ time series components: $m_{c,d}(k), m_{c,d}(k-1), \dots , m_{c,d}(k-l), \dots , m_{c,d}(k-(N_l-1))$. Consequently, for the $N_d$ variables measured from the $N_c$ vehicles, each variable is expanded in the time direction, leading to $N_l$ augmented measurement data points. The reservoir state is then defined as $x \in \mathbb {R}^{N_x}$, where $N_x = N_c \times N_d \times N_l$. Using the reservoir state *x*(*k*) obtained through this procedure, the predicted value $\hat{y}(k+1)$ is calculated using Eq. (4).

4 $$\begin{aligned} \hat{y}(k+1) = \sum ^{N_c}_{c=1} \sum ^{N_d}_{d=1} \sum ^{N_l-1}_{l=0} W^{\text {out}}_{c,d,l} m_{c,d}(k\tau -l) = W^{\text {out}}x(k) \end{aligned}$$

Because the actual measured variables are velocity, acceleration, and power consumption, the number of observation variables is $N_d = 3$. Furthermore, with a step interval of $\tau = 5$ s, the maximum number of time direction data points which can be provided to the reservoir at each step is set as $N_l = \tau = 5$. The readout weights $W^{\text {out}}$ are obtained using ridge regression, as in conventional RC, following Eq. (3). The schematic diagram of this computational framework for a scaled traffic system is presented in Fig. 4.

### Grid road traffic simulation

We implement a multi-agent simulation in which vehicles travel on the grid road network to reproduce traffic observed in real transportation systems. Because the numerical simulation does not incorporate physical conditions such as friction or hardware constraints, it differs fundamentally from physical experiments. However, it allows for more flexible parameter settings and overcomes the limitations of scaled model experiments, such as the restricted number of vehicles and the increased time required for experimentation. Consequently, it supports more detailed investigations. We configure a $2 \times 3$ grid road network, as presented in Supplementary Figure B1. Each vehicle obeys traffic signals and avoids collisions with the vehicle ahead, as in the real world. Similarly to the scaled traffic experiments, the system has no vehicle inflow or outflow. Therefore, the total number of vehicles $N_c$ remains constant. Each road has two lanes (opposing directions). All roads share a common length of $L = 50$. Unlike the scaled traffic course, traffic lights are installed at every intersection. All traffic lights synchronize. At the T-shaped intersections labeled Intersection 1 and Intersection 4 in Supplementary Figure B1, vehicles randomly choose their direction of travel each time they pass through. Here, $N_r$ denotes the total number of roads. $N_i$ stands for the number of signalized intersections. For this simulation, we have $N_r = 14$ and $N_i = 6$.

The measurement data obtained from the traffic simulation consist of the number of vehicles $m_r$ traveling on each road at a given time, and the number of vehicles $m_i$ passing through each intersection per unit of time. The specific procedure for formatting the measurement data $m_r, m_i$ as the reservoir state *x* is the following. At time step *k*, the number of vehicles measured on each road $r = 1,2, \dots , N_r$ is represented as $m_r(k)$. The passing traffic measured at each intersection $i = 1,2, \dots , N_i$ is represented as $m_i(k)$.

Variable $m_r$, which represents the number of vehicles on each road segment, exhibits slower dynamics over time compared to variable $m_i$, which represents the flow at intersections. This difference arises because $m_r$ reflects traffic variations distributed over an entire road, whereas $m_i$ captures localized and more immediate changes at intersections. Therefore, for $m_r$, past values over $N_l$ time steps, specifically $m_r(k), m_r(k-1), \dots , m_r(k-l), \dots , m_r(k-(N_l-1))$, are incorporated into the reservoir state *x*(*k*) at time step *k*: the method of incorporating temporal information differs from that in the scaled traffic system. Furthermore, $m_r$ and $m_i$ change in a discrete manner. To make them more suitable for continuous time-series prediction, these variables are smoothed, respectively, for each road and intersection $\tilde{m}_r, \mathrm{and} \,\tilde{m}_i$. They are then subjected to a slight nonlinear transformation to obtain $g(\tilde{m}_r)$ and $g(\tilde{m}_i)$. Details of this transformation, together with illustrative examples, are provided in the Supplementary Information (Section B).

**Fig. 5:**
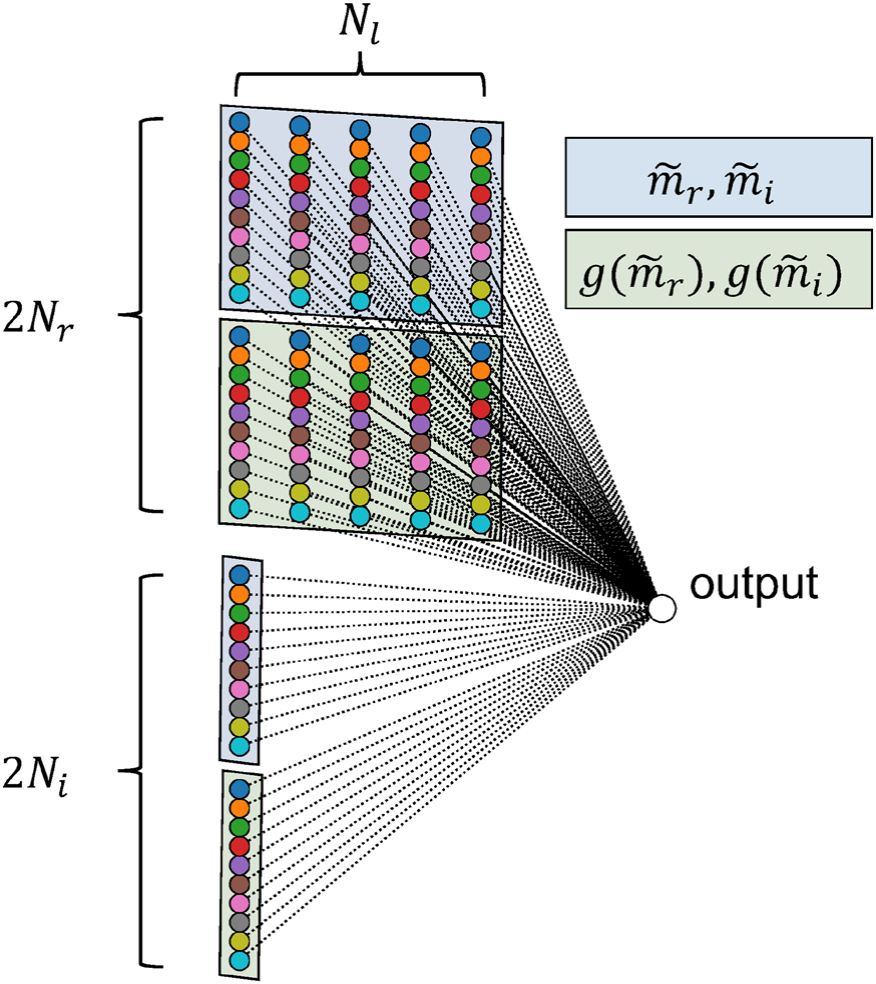
Schematic showing the Road Traffic Reservoir Computing (RTRC) model for the grid traffic simulation.

Based on the procedures described above, the reservoir state $x \in \mathbb {R}^{N_x}$ is defined as $[\tilde{m}_{r,l}(k); \tilde{m}_i(k); g(\tilde{m}_{r,l}(k)); g(\tilde{m}_i(k))]$, where $N_x = 2N_r \times N_l + 2N_i$, and where $[\cdot ;\cdot ]$ stands for a vector concatenation. The predicted value is then obtained using Eq. (5). Here, $W^{\text {out}}_{r,l,*}$ and $W^{\text {out}}_{r,l,g}$ denote the readout weights associated with the smoothed and nonlinear transformed observations of road *r* at *l*, whereas $W^{\text {out}}_{i,*}$ and $W^{\text {out}}_{i,g}$ respectively denote those for intersection *i*. Then $W^{\text {out}}$ is obtained using ridge regression with Eq. (3), as in conventional RC and the scaled traffic system model. The delay step is set to $N_l = 20$. A conceptual diagram of this computational framework for traffic simulation is presented as Fig. 5.

5 $$\begin{aligned} \hat{y}(k+1)= & \sum ^{N_r}_{r=1} \sum ^{N_l-1}_{l=0} \Big ( W^{\text {out}}_{r,l,*} \tilde{m}_{r}(k-l) + W^{\text {out}}_{r,l,g} g(\tilde{m}_{r}(k-l)\Big )\nonumber \\ & + \sum ^{N_i}_{i=1} \Big ( W^{\text {out}}_{i,*} \tilde{m}_{i}(k) + W^{\text {out}}_{i,g} g(\tilde{m}_{i}(k)\Big )\nonumber \\ = & W^{\text {out}}x(k) \end{aligned}$$

## Results

For the HRC proposed herein, the goal is to predict traffic states in scaled model experiments and to approximate nonlinear functions in numerical simulations based on traffic light patterns directly by extracting information from observed dynamics, rather than using intentionally designed reservoirs. In the case of RTRC, where traffic flow is used as the reservoir, the reservoir characteristics (i.e., traffic flow) vary depending on the traffic density. The following experiments, by elucidating the relation between the traffic density and accuracy, clarify the density range within which road traffic works effectively as the reservoir.

First, we use a scaled traffic system that reproduces traffic flow on a test bed to predict internal traffic states. Next, as an example of treating road traffic as the physical reservoir, we conduct a function approximation task in a simulation using temporal traffic light patterns as inputs. Furthermore, we perform a delay task to measure memory capacity: a typical performance indicator of RC. This approach then provides a basis for discussing the conditions under which HRC can be established.

### Prediction for scaled traffic experiments

**Fig. 6:**
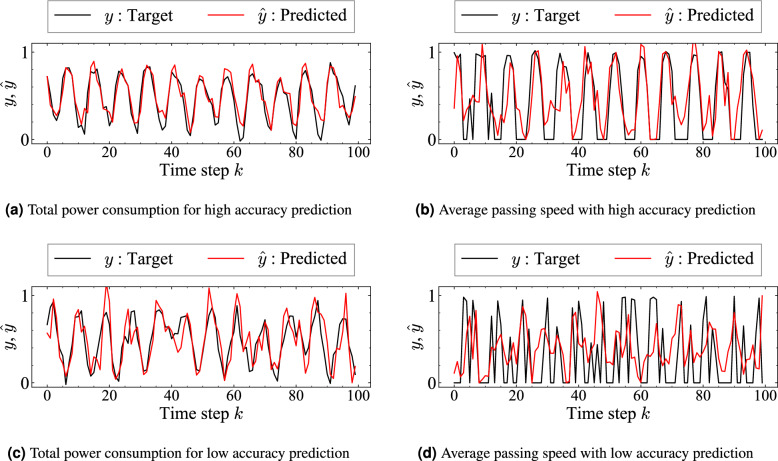
Prediction results in the scaled traffic experiments. (**a**) Total power consumption in the case of high-accuracy prediction with $N_c = 6$, where the predicted values (red) closely follow the ground-truth target (black). (**b**) Average passing speed in the case of high-accuracy prediction with $N_c = 8$, showing good agreement between the target and prediction. (**c**) Total power consumption in the case of low-accuracy prediction with $N_c = 10$, where the deviation between the prediction and the target becomes more pronounced. (**d**) Average passing speed in the case of low-accuracy prediction with $N_c = 4$, also exhibiting larger discrepancies than those of the high-accuracy case.

**Fig. 7:**
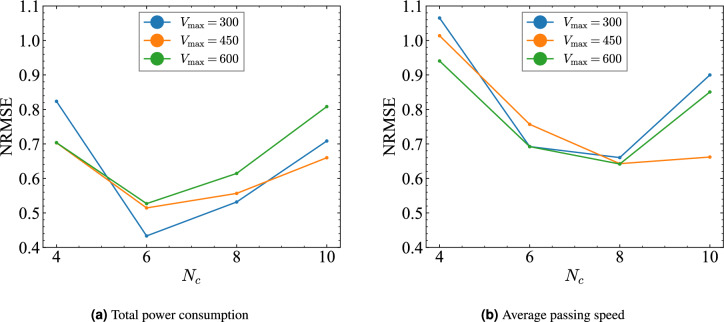
Prediction error with respect to the number of vehicles $N_c$ in the scaled traffic experiments. (**a**) NRMSE of total power consumption for $V_{\max } = 300, 450, 600$. (**b**) NRMSE of the average passing speed under the same conditions.

In scaled traffic experiments, the relation between the number of vehicles, which corresponds to traffic density, and prediction accuracy is evaluated using the prediction task. The prediction targets are (I) the total power consumption of all vehicles (total consumption power), and (II) the average passing speed at a specific location within the course. Both are predictions made at one time-step ahead. The measurement location for the average passing speed in the prediction task (II) is the point before Intersection A, which is marked as green in Fig. 2a . The training dataset and test dataset respectively consisted of 200 time steps and 100 time steps. The initial positions of the vehicles were assigned randomly. Measurements were taken after transient effects had dissipated. The regularization coefficient was set as $\beta = 10$, chosen as a representative value. Results for other $\beta$ values are presented in Supplementary Table C1. All time series data used as training data were normalized per variable. The test data were scaled using the same scale as the training data normalization. The accuracy of time series prediction was evaluated using the normalized root mean square error ($\text {NRMSE}$), as calculated by dividing the root mean square error ($\text {RMSE}$) between the target values *y* and the predicted values $\hat{y}$ in the test data by the standard deviation of *y*.

Figure 6a shows prediction results obtained when the maximum speed of all vehicles was set as $V_{\max } = 300$ [mm/s] and the number of vehicles was $N_c = 6$, while Fig. 6b shows the results for $N_c = 8$. The results indicate that the prediction model learns the trend of the target time series successfully, demonstrating the feasibility of the predictive model. For task (II), negative predicted speed values are replaced with zero because the vehicle speed cannot be negative. Also, Figs. 6c and 6d show examples in which prediction is performed on measurement data from: (I) a high-density scenario with $N_c = 10$ for total power consumption prediction and (II) a low-density scenario with $N_c = 4$ for average passing speed prediction.

Figures 7a and  7b show the relation between the number of vehicles $N_c$ and $\text {NRMSE}$ for each prediction task under different maximum vehicle speeds $V_{\text {max}} = 300, 450, 600$ [mm/s]. In the scaled model experiments, $V_{\text {max}}$ represents the maximum speed which vehicles can reach when no obstacle is present. The procedure for setting vehicle speed regulation is described in the Supplementary Information (Section A). The results indicate that, irrespective of $V_{\text {max}}$, the prediction accuracy improves at intermediate densities ($N_c = 6$ or 8), but it deteriorates at both low ($N_c = 4$) and high ($N_c = 10$) traffic densities. This difference in prediction accuracies suggests the existence of an optimal density regime in which RTRC reaches its highest computational performance. In addition, in the scaled traffic system, variations in $V_{\text {max}}$ have little effect on prediction accuracy: vehicles seldom reach their maximum speeds because of the course layout, the course size, and the effects of traffic lights.

#### Stability analysis for the scaled traffic experiment

To examine the dynamical stability of the harvested reservoir states, we analyzed the observed time-series data obtained from the scaled traffic experiment. The state dimensionality was defined as $N_x = N_c N_d N_l$ with $N_d = 3$ and $N_l = 5$, resulting in $N_x \in \{60, 90, 120, 150\}$ for $N_c = \{4,6,8,10\}$. Because all measured variables are physically constrained by vehicle control limits and course geometry, the trajectories *x*(*k*) evolve on a compact set $\mathcal {X}_{\textrm{scaled}}\subset \mathbb {R}^{N_x}$.

The maximal Lyapunov exponent (LLE) and the mean local growth rate $\lambda _{\textrm{avg}}$ were estimated from the recorded state trajectories to quantify the divergence of nearby trajectories in the state space. The analysis procedure and parameter settings are summarized in the Supplementary Information (Section C). Table 1 reports the representative case for $V_{\max }=300~\mathrm {mm/s}$.

**Table 1:** Mean local growth rate $\lambda _{\textrm{avg}}$ (per step) in the scaled traffic experiment ($V_{\max }=300~\mathrm {mm/s}$).

Number of vehicles $N_c$	Dimensionality $N_x$	$\lambda _{\textrm{avg}}$ (per step)
4	60	4.09
6	90	2.70
8	120	2.18
10	150	1.85

The mean growth rate decreased monotonically as the number of vehicles increased, indicating that the observed dynamics became progressively more reproducible at higher densities. Importantly, the lowest divergence occurred at the highest density ($N_c=10$), whereas the best prediction accuracy appeared at medium densities ($N_c=6,8$; see Fig. 7). This observation suggests that RTRC performance benefits from a balance between reduced divergence (stability) and sufficient nonlinear responsiveness, which is achieved before the divergence reaches its minimum.

#### Baseline comparison and real-time performance

To objectively assess both performance and computational efficiency, we conducted benchmark experiments under the same conditions as the scaled traffic experiments ($N_c = 6$, $V_{\max } = 300$). Three representative sequence-learning models were adopted as baselines: the Echo State Network (ESN), a small Long Short-Term Memory (LSTM) network, and the autoregressive integrated moving average model with exogenous regressors (ARIMAX). All models were trained and evaluated on the same preprocessed input and target sequences; for LSTM, a fixed window length was derived from the same sequences. For each model, hyperparameters were tuned within predefined ranges to achieve the best accuracy based on validation NRMSE (see the Supplementary Information (Section C) for details). The primary metric was NRMSE for both the total power consumption and average passing speed prediction tasks.

Table 2 summarizes the results. For power consumption, the proposed method achieved the lowest error (NRMSE = 0.392), outperforming LSTM (0.482), ESN (0.531), and ARIMA (2.621). For average speed, LSTM obtained the lowest NRMSE (0.667), while RTRC was nearly identical (0.683) and substantially better than ESN and ARIMA. These results indicate that RTRC achieves the best accuracy for power prediction and remains competitive with LSTM for average-speed prediction.

We also measured training time and per-step inference latency under identical settings. RTRC required approximately 0.15 ms for training and 2.2 $\mu$s for per-step inference; ESN required 5–10 ms and 25–50 $\mu$s, LSTM about 300 ms and 68 $\mu$s, and ARIMA 3–5 s and about 1 ms, respectively. Thus, RTRC was several orders of magnitude faster in both training and inference. Given a physical update period of $\Delta t^{*} = 5$ s, the real-time factor $\textrm{RTF} = \Delta t^{*} / L_{\textrm{step}}$ is approximately $2.3 \times 10^{6}$, showing that RTRC easily satisfies real-time constraints. The measured latencies and training times are consistent with the detailed evaluation in Section C of the Supplementary Information (1.6–1.7 $\mu$s for inference and 0.9 ms for training at $N_c = 10$), supporting consistency across configurations.

These findings demonstrate that, compared with conventional sequence-learning models, RTRC offers a markedly better trade-off between accuracy and real-time capability. Although LSTM achieved slightly lower error on average-speed prediction, RTRC attained nearly comparable accuracy while being orders of magnitude faster in both training and inference. For power consumption prediction, RTRC delivered the best accuracy together with the smallest computational cost, supporting its suitability for real-time operation and feasible online adaptation.

The computational times in Table 2 reflect the energy consumption of each model during execution. Since the average power draw of the CPU remains nearly constant across models under identical hardware conditions, a shorter processing time corresponds to lower energy usage. Because RTRC is several orders of magnitude faster than the other models in both training and inference, it also exhibits the smallest computational energy consumption. Therefore, RTRC outperforms the baseline models not only in accuracy and computational efficiency but also in energy efficiency.

**Table 2:** Comparison of NRMSE and computational costs across models ($N_c = 6$, $V_{\max } = 300$). Upper: total power consumption prediction; lower: average passing speed prediction. Values are means; jitter statistics are reported in the Supplementary Information (Section C).

Model	NRMSE	Training [ms]	Inference [$\mu$s]
**Total power consumption prediction**			
RTRC	0.392	0.15	2.20
ESN	0.531	5.28	24.77
LSTM	0.482	319.99	67.80
ARIMA	2.621	3263.48	977.75
**Average passing speed prediction**			
RTRC	0.683	0.16	2.22
ESN	0.950	9.87	53.29
LSTM	0.667	303.22	67.01
ARIMA	1.349	4775.36	1164.72

### Memory capacity evaluation for grid road traffic simulation

**Fig. 8:**
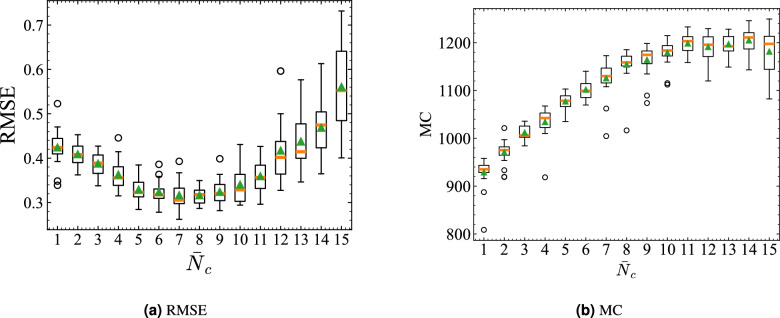
Model performance metrics as a function of the number of vehicles per road $\bar{N}_c$ in numerical simulations. (a) $\text {RMSE}$ for a nonlinear function approximation task. (b) Memory capacity ($\text {MC}$), indicating how well the system retains past input information. Results are averaged over 20 trials with random initial configurations. Boxplots show the distribution of results: The orange horizontal line represents the median. The green triangle denotes the mean. The box indicates the interquartile range. Whiskers and circles represent variation outside the upper and lower quartiles.

Performance in the scaled traffic system experiment relies only on prediction error, which makes it necessary to evaluate RTRC using conventional RC metrics. Therefore, we measure the short-term memory of the road traffic reservoir using memory capacity ($\text {MC}$)^68^, a metric that quantifies how well the reservoir retains past input information. For the traffic simulation, we conducted a function approximation task to assess the accuracy of approximating a nonlinearly transformed input sequence *f*(*s*(*k*)). Then we evaluated $\text {MC}$ through a delay task that measures how well past input values can be reconstructed. Additional details on $\text {MC}$ and the delay task are provided in the Supplementary Information (Section B).

#### Function approximation task

For the function approximation task, the target is *f*(*s*(*k*)). Eq. (5) is used not as a prediction model but as a function approximation model. Measurement data $m_r$ and $m_i$ used for training and testing consist of 2000 time steps each. The regularization coefficient is set as $\beta = 10$. Function approximation accuracy is evaluated using RMSE and is averaged over 20 training and testing pairs.

The average number of vehicles per road in the entire network is defined as $\bar{N}_c$. For example, when $\bar{N}_c = 1$, the total number of vehicles $N_c = \bar{N}_c \times N_r = 1 \times 14 = 14$. Consequently, $\bar{N}_c$ represents the average traffic density per single road (one direction) in the grid network. Figure 8a presents results of 20 trials in which the initial positions were assigned randomly. The results indicate that $\text {RMSE}$ takes lower values when $\bar{N}_c = 7, 8, 9, 10$. If this range is regarded as medium density, then errors can be expected be higher at both lower and higher densities. Larger deviations from the medium-density region engender greater errors. For large values of $\bar{N}_c$, the initial positions of vehicles caused density variations among roads, which could not be eliminated completely and which caused greater variation in approximation accuracy. Therefore, consistent with the scaled traffic experiments, the simulation results suggest that the proposed numerical model performs optimally in a certain range that corresponds to medium density.

#### Delay task

The measured values of $\text {MC}$ for each $\bar{N}_c$ in the delay task are demonstrated. First, $\text {MC}_\tau$ in Eq. (B1) of the Supplementary Information (Section B) is calculated using a dataset of 4000 time steps, with the maximum delay step set as $\tau _{\max } = 2000$ in Eq. (B2). Consequently, the maximum possible value of $\text {MC}$ is 2000. Figure 8b presents the average $\text {MC}$ over 20 trials for each $\bar{N}_c$, where the initial positions of vehicles are assigned randomly. In the low-density region, $\text {MC}$ is small, but it increases as $\bar{N}_c$ grows, suggesting that, with fewer vehicles, the memory of past traffic states is lost easily. As the number of vehicles, or traffic density, increases, road states are more likely to be retained. However, although $\text {MC}$ increases with density up to the medium-density range, it tends to saturate or even decrease in the high-density region. This tendency suggests that the road capacity, as determined by the road length *L*, constrains $\text {MC}$.

To further evaluate the statistical reliability of the estimated $\text {MC}$ under a finite sequence length, a circular block bootstrap analysis was performed for a representative seed. This resampling method divides the time series into contiguous blocks based on the autocorrelation length and reconnects them circularly, thereby preserving short-term temporal correlations while assessing finite-sample variability. Under the condition of 4000 time steps and $\tau _{\max }=2000$, with 100 bootstrap resamples, the resulting 95% confidence intervals of the total $\text {MC}$ were within $\pm 4$–$5\%$ of the mean, and the relative standard deviation was approximately 2–$3\%$. These results confirm that the estimated $\text {MC}$ is statistically stable and that the density-dependent trend and the optimal region in Fig. 8b are not affected by finite-sample fluctuations (see the Supplementary Information, Section B, for details).

#### Evaluation under random phase offsets

To assess whether intersection-wise input synchronization affects computational performance, we introduced fixed integer phase offsets to the traffic light inputs at each intersection and evaluated the reservoir under asynchronous inputs. In our traffic simulation, each intersection’s signal sequence $s_i(k)$ is generated as a random piecewise-constant binary process (see the Supplementary Information, Section B, for details). In the baseline configuration, all intersections reference the same random sequence. Here, for the asynchronous setting, we assigned a fixed offset $\phi _i$ drawn uniformly from the integer range $[-5,+5]$ (in time steps) to each intersection and read the input as $s_i(k+\phi _i)$ with boundary clipping. This range corresponds to at most roughly 10% of the minimum block length and thus desynchronizes intersections without altering the input run-length statistics.

To isolate the effect of input desynchronization from the initial traffic configuration, we fixed a single initial vehicle positions and conducted 20 trials by varying only the random seeds that determine $\phi _i$. As a reference, we also evaluated a synchronized run ($\phi _i=0$) under the same initial state. We report results at $\bar{N}_c=7$, for which the baseline median RMSE attained its minimum among the settings we explored.

**Fig. 9:**
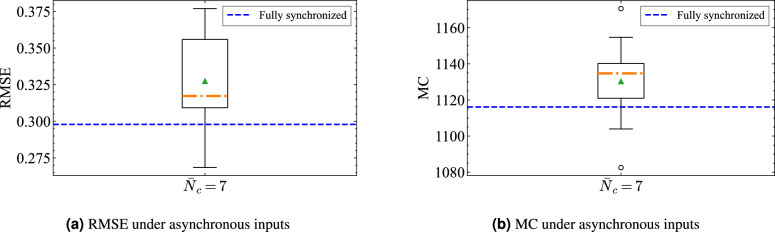
Performance under random phase offsets at intersections, evaluated at $\bar{N}_c=7$ (the setting with the smallest baseline median RMSE). Boxplots summarize 20 trials with different random seeds for fixed integer offsets $\phi _i \in [-5,+5]$ applied per intersection, while the horizontal dashed line indicates the result for the fully synchronized case ($\phi _i=0$) using the same initial traffic state. Relative to the asynchronous median, the synchronized baseline differed by approximately $+6.5\%$ in RMSE and $+1.7\%$ in MC. The dot–dashed line shows the median, and the triangle indicates the mean.

Figure 9 summarizes the outcomes for the two primary metrics used throughout this study: RMSE and MC. The distributions for the asynchronous condition are shown as boxplots, and the synchronized result for the same initial state is overlaid as a horizontal dashed line. Relative to the asynchronous median, the synchronized baseline differed by approximately $+6.5\%$ in RMSE and $+1.7\%$ in MC. In both metrics, the synchronized value lies near the inter-quartile range of the asynchronous distribution, indicating that the approximation capability and short-term memory are slightly getting worse but not significantly varied under input de-synchronization.

#### Evaluation under link-length heterogeneity

To examine whether the proposed method remains effective in road conditions that are not perfectly regular, we evaluated the computational performance when heterogeneity in the road length *L* was introduced. The traffic density was fixed at the medium-density condition of $\bar{N}_c = 7$. Specifically, a $2\times 4$ grid network was constructed, and one of the vertical roads was completely closed so that vehicles could not travel along that section. Consequently, although the nominal structure remained $2\times 4$, the absence of the central vertical road made the network function effectively as a $2\times 3$ grid. The intersections connected to the removed road were retained, and their traffic signals continued to operate synchronously with the other intersections. As a result, the distances between intersections varied from those in the uniform grid, forming a nonuniform topology in which the effective length of each road differed. A snapshot of vehicle movements on this effectively $2\times 3$ network is illustrated in Supplementary Figure B2.

Under this network with heterogeneous road lengths, the function approximation error was $\text {RMSE}=0.4043$, and the memory capacity was $\text {MC}=1359.16$ ($\tau _{\max }=2000$). In comparison, the default condition shown in Figs. 8a , 8b for $\bar{N}_c=7$ yielded an average $\text {RMSE}=0.3159$ and $\text {MC}=1125.18$. Although the RMSE slightly increased, the MC expanded by approximately 1.21 times, indicating that the heterogeneity in road length enhanced the system’s ability to retain memory. This suggests that spatial variations in road length, and hence in local traffic capacity, can make the memory capacity larger. These results confirm that the proposed method remains effective even for road networks with nonuniform link lengths, demonstrating robustness beyond perfectly regular grid structures.

#### Realistic routing and open boundary conditions

To consider more realistic traffic conditions, we conducted a $6\times 6$ grid traffic simulation. By extracting the central $2\times 2$ grid, the internal links corresponding to this region were treated as an open subsystem of the overall network, and both the function approximation error and the memory capacity (MC) were evaluated. The traffic density was fixed at the medium-density condition of $\bar{N}_c = 7$.

Routing was implemented as a softmax-based probabilistic model to emulate realistic driving behavior. At each intersection, the vehicle’s approach direction was defined according to the intersection geometry, and the baseline probability vector for the three possible maneuvers–straight, right, and left–was defined as

$$\textbf{p}^{(0)} = \bigl (p^{(0)}_{\textrm{st}},\, p^{(0)}_{\textrm{rt}},\, p^{(0)}_{\textrm{lt}}\bigr ).$$

The softmax routing model was applied to all intersections in the network. For the intersections located at the center $2\times 2$ grid, which correspond to the main traffic corridor, the baseline probabilities were set to $\textbf{p}^{(0)}=(0.70, 0.15, 0.15)$ for vehicles approaching from the vertical direction and $\textbf{p}^{(0)}=(0.65, 0.20, 0.15)$ for vehicles approaching from the horizontal direction, reflecting the tendency that drivers prefer to go straight. For the remaining intersections, where no specific routing preference was imposed, an equal baseline probability of $(1/3,\,1/3,\,1/3)$ was used. These baseline values were converted into logarithmic scores and dynamically adjusted according to the signal state, the local congestion level, and temporal variations in traffic demand. Specifically, the logit value for maneuver $\alpha \in \{\textrm{st}, \textrm{rt}, \textrm{lt}\}$ at simulation step *k* was defined as

$$z_\alpha (k) = \log \!\bigl (p^{(0)}_{\alpha } + \varepsilon \bigr ) + \Delta _{\textrm{sig},\alpha }(k) + \Delta _{\textrm{cong},\alpha }(k) + \Delta _{\textrm{time},\alpha }(k),$$

where $\varepsilon = 10^{-9}$ is a small constant for numerical stability. The signal term $\Delta _{\textrm{sig},\alpha }(k)$ decreases the logit (approximately by $-10$) when the corresponding signal is red. The congestion term $\Delta _{\textrm{cong},\alpha }(k)$ slightly decreases the logit in proportion to the recent inflow count $N_{\textrm{in},\alpha }(k)$, and the temporal term $\Delta _{\textrm{time},\alpha }(k)$ introduces diurnal demand fluctuation modeled as $\Delta _{\textrm{time},\alpha }(k) = A \sin (2\pi k / K_T)$ with amplitude $A=0.10$ and period $K_T=3600$ simulation steps. The composite logit vector $\textbf{z}(k)$ was then normalized by the softmax function to yield the selecting probability for each direction:

$$p_\alpha (k) = \frac{\exp (z_\alpha (k))}{\sum _{\beta \in \{\textrm{st,rt,lt}\}}\exp (z_\beta (k))}.$$

This formulation allows the probability distribution to vary smoothly according to the signal color, local congestion, and temporal demand, resulting in realistic routing behavior–for example, vehicles tend to avoid going straight when facing a red light and to choose less congested directions when local traffic density increases.

Under these conditions, when observing only the internal links of the central $2\times 2$ grid, the function approximation error was $\text {RMSE}=0.3607$, indicating high accuracy under the fixed medium-density condition. The memory capacity was $\text {MC}=784.17$ ($\tau _{\max }=2000$), which was smaller than that obtained when observing the entire network; however, even when the observation range was restricted to a subnetwork, short-term memory was still observed.

## Discussion

**Fig. 10:**
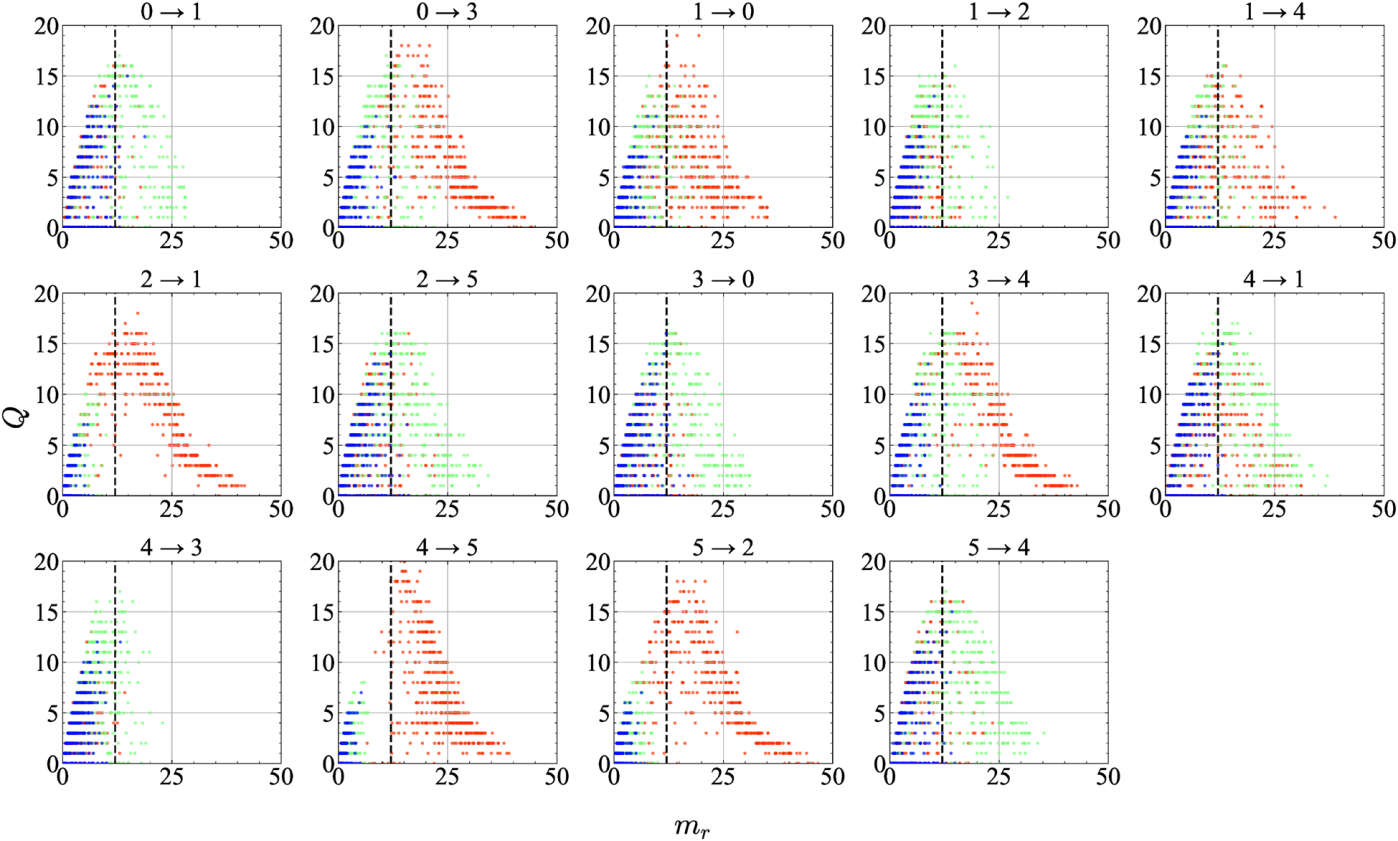
Fundamental diagrams for each road segment in the traffic simulation, showing the relation between the number of vehicles $m_r$ and the traffic flow *Q* per 100 time steps. Three density settings are compared: $\bar{N}_c = 3$ (blue), 8 (green), and 13 (red). Each subplot represents a specific road segment. The dashed vertical line represents the critical vehicle count $\bar{N}^{\rho _c}=12$, as derived from the stability condition of the optimal velocity model.

**Fig. 11:**
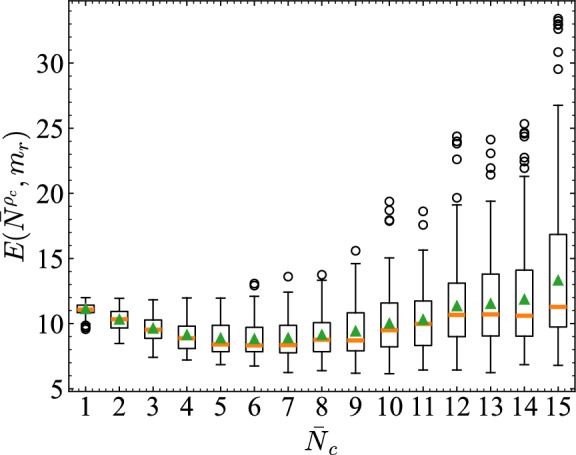
Deviation from the critical vehicle count $\bar{N}^{\rho _c}$ across different densities $\bar{N}_c$. The error is computed as the root mean square error between the observed vehicle count $m_r$ and the critical value. Box plots represent distributions across all road segments and 20 simulation trials.

**Fig. 12:**
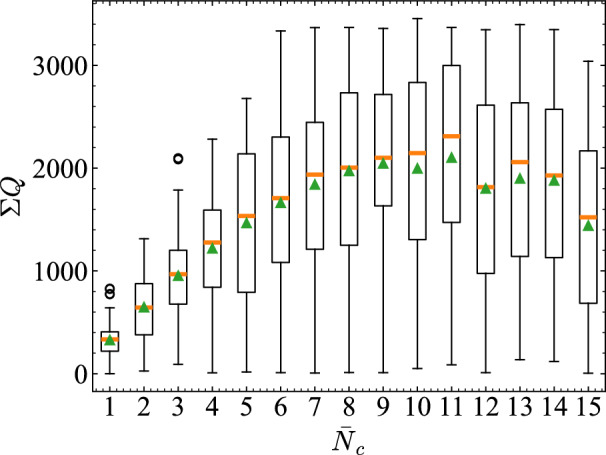
Total traffic flow $\Sigma Q$ accumulated over time across different densities $\bar{N}_c$. Results are shown as box plots across 20 trials, thereby illustrating that the medium-density regime achieves the highest average throughput.

**Fig. 13:**
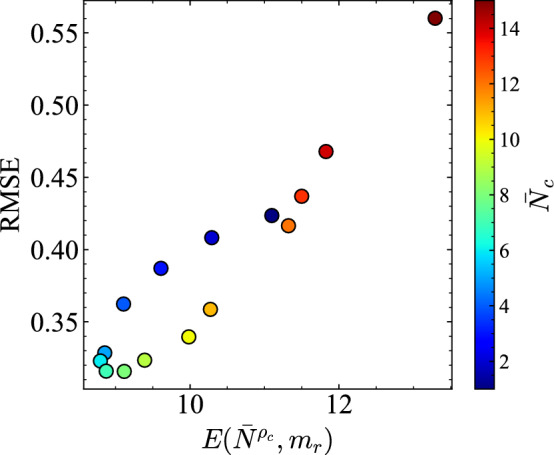
Relation between deviation from critical density $E(\bar{N}^{\rho _c}, m_r)$ and approximation error $\text {RMSE}$. Each point corresponds to the result obtained at a specific $\bar{N}_c$. Also, $\text {RMSE}$ reaches its minimum when the deviation from the critical density is minimized.

**Fig. 14:**
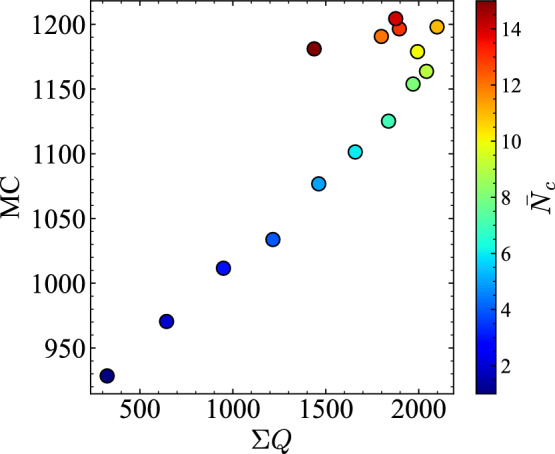
Scatter plot showing the relation between total traffic flow $\Sigma Q$ and memory capacity ($\text {MC}$) for varying $\bar{N}_c$. A near-linear increase is observed in the low-to-medium density range, with subsequent saturation in high-density conditions.

For the medium-density region where the computational performance of the RTRC is maximized, it is suggested that the reservoir dynamics are interpreted as achieving a balance between linearity and nonlinearity. Regarding traffic flow dynamics, traffic is well known to exhibit two distinct regimes depending on density: free flow and congested flow^61–63^. Moreover, it has been speculated that the characteristics of these dynamics might change with density according to the strength of nonlinear interactions among vehicles. Based on this understanding, we assess the relation between the density range with high accuracy and the occurrence of traffic congestion.

A fundamental diagram portrays the relation between traffic flow and road density. Distinct traffic characteristics are observed before and after the critical density^69,70^. Figure 10 depicts the fundamental diagram of traffic for each road, based on simulation data collected over 45,000 time steps after excluding the initial 5,000 time steps. The diagrams show three cases, $\bar{N}_c = 3, 8, 13$, presented respectively in blue, green, and red. Here, the vertical axis denotes traffic flow *Q* per 100 time steps. The horizontal axis denotes the number of vehicles $m_r$ per road over 100 time steps. Each panel labeled “a $\rightarrow$ b” depicts a road from Intersection A to Intersection B. The dashed black line shows the critical vehicle count $\bar{N}^{\rho _c} = 12$, derived from the stability condition of the optimal velocity model, $a > 2V_{\text {opt}}'(h)$^35,71^, with sensitivity $a = 5$.

The results show that, as in real traffic, when the number of vehicles on a road $m_r$ is near the critical value $\bar{N}^{\rho _c}$, traffic flow *Q* is high. However, *Q* decreases as $m_r$ deviates from $\bar{N}^{\rho _c}$. Consequently, exceeding $\bar{N}^{\rho _c}$ indicates the onset of congestion on that road. Comparison across densities shows that, in the low-density case ($\bar{N}_c = 3$), $m_r$ rarely exceeds $\bar{N}^{\rho _c}$. In the high-density case ($\bar{N}_c = 13$), some roads markedly exceed $\bar{N}^{\rho _c}$, whereas others carry fewer vehicles than in the low-density case, indicating that uneven initial distributions engender greater variance in road densities. In the medium-density case ($\bar{N}_c = 8$), both congested and uncongested roads coexist, which indicates that high-accuracy computation is possible when road densities are maintained near the congestion threshold.

Based on Fig. 10, we quantitatively evaluated the relation between $\bar{N}_c$ and congestion. Figure 11 shows the deviation between $m_r$ and $\bar{N}^{\rho _c}$, calculated as the $E(\bar{N}^{\rho _c}, m_r)=\sqrt{\frac{1}{n}\sum _{k=1}^{n}(m_r-\bar{N}^{\rho _c})^2}$. The box plots show distributions across roads and 20 trials. Compared to low-density and high-density traffic, the medium-density regime exhibits lower values of $E(\bar{N}^{\rho _c}, m_r)$ and smaller variance, suggesting that vehicle counts on roads tend to remain close to $\bar{N}^{\rho _c}$. Figure 12 presents the total traffic volume $\Sigma Q$ over time for each $\bar{N}_c$. $\Sigma Q$ is defined as the sum of the traffic flows *Q* over all roads. Specifically, the number of vehicles passing through each road cross-section was counted by dividing the simulation into intervals of 100 time steps. This number was inferred as the traffic flow *Q*.

As with Fig. 11, the box plots summarize distributions across roads and trials. Traffic flow increases with $\bar{N}_c$ up to medium density, but it then decreases slightly in high-density conditions. Overall, these results suggest that the medium-density region, where RTRC shows high prediction accuracy, corresponds to a state in which densities cluster near the congestion threshold and throughput is maximized.

Next we present consideration of how the prediction error RMSE and $\text {MC}$ relate to $E(\bar{N}^{\rho _c}, m_r)$ and $\Sigma Q$ across density settings. Figure 13 shows that RMSE is minimized when deviations from the critical density are smallest, particularly at $\bar{N}_c = 6, 7$. Figure 14 shows the relation between $\Sigma Q$ and $\text {MC}$. Both increase approximately linearly from low to medium density, but saturate around $\bar{N}_c = 8, 9$, which indicates that the computational performance depends strongly on conditions near the transition point to congestion and on the high throughput of traffic flow. Although traffic is not designed explicitly to operate near $\bar{N}_c$, Fig. 10 suggests that, in the high-accuracy medium-density regime, a state naturally emerges with coexistent pre-congestive dynamics and post-congestive dynamics.

To quantitatively support these interpretations, we analyzed the statistical relationships between RMSE and $E(\bar{N}_{\rho _c}, m_r)$, and between $\text {MC}$ and $\Sigma Q$ across different density ranges. For the RMSE–*E* relation shown in Fig. 13, the Spearman correlation coefficient was $\rho = 1.00$ in the low-density regime ($\bar{N}_c = 1$–6), indicating that error reduction follows the increase in density. In contrast, in the medium-to-high-density regime ($\bar{N}_c = 7$–15), the correlation remained strong ($\rho = 0.983$), but as seen in the figure, a nonlinear dependence emerged in which RMSE sharply decreases when *E* is small.

For the $\text {MC}$–$\Sigma Q$ relation shown in Fig. 14, a tight coupling was observed in the low-to-medium density range, with a high Pearson correlation coefficient of $r = 0.997$. This indicates that memory capacity increases almost linearly with throughput until saturation. At higher densities ($\bar{N}_c = 10$–15), however, this relationship weakened ($r = 0.42$), suggesting that congestion suppresses information propagation.

**Fig. 15:**
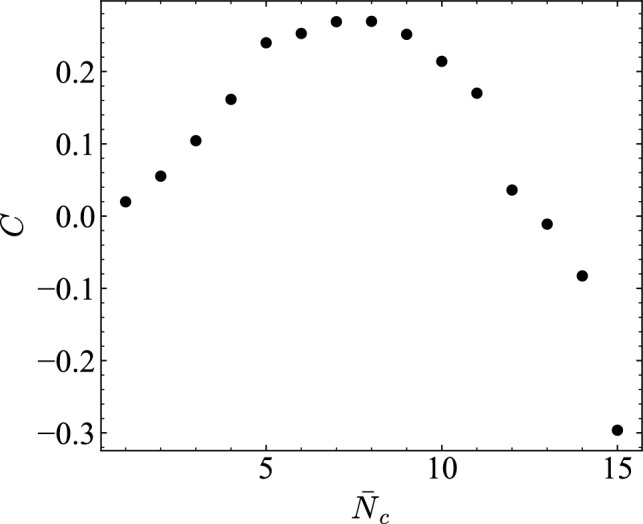
Empirical information-processing capacity (IPC) $C = 1 - (\textrm{RMSE}/\textrm{std}(z))^2$ as a function of the average number of vehicles per road $\bar{N}_c$. The capacity increases with density and peaks in the medium-density regime ($\bar{N}_c \approx 7$–9), consistent with IPC theory. Negative values at high densities suggest a loss of sensitivity to input variations due to traffic saturation.

In the present Road Traffic Reservoir Computing (RTRC) framework, the observed traffic flow is interpreted as a dynamical system *X* with state variables $\{x_i(t)\}$, and the system is formalized in the same manner as standard reservoir computing by training a linear readout on the observed states. According to the Information-Processing Capacity (IPC) theory ^60^, the capacity $C[X,y_\ell ]$ is defined as the ability of a system to reconstruct an orthogonal set of basis functions $\{y_\ell \}$ of the input sequence, and the total capacity is theoretically upper-bounded by the number *N* of linearly independent observable variables. When the system exhibits fading memory properties, this upper bound is saturated, and the total capacity can be decomposed into linear memory and nonlinear computational components that exhibit a trade-off between memory and nonlinearity.

Note that, the capacity *C* defined in this study differs from the total capacity strictly defined in IPC theory for simplicity. It is evaluated using the accuracy of a function approximation task in the grid road traffic simulation for a single nonlinear function $z=f(s(k))$. Specifically, by applying the IPC formulation, $C[X,z] = 1 - \frac{\textrm{MSE}(\hat{z}, z)}{\textrm{Var}(z)},$ the value of *C* was computed from the ratio of RMSE to the standard deviation of *z* as

$$C = 1 - \left( \frac{\textrm{RMSE}}{\textrm{std}(z)}\right) ^2.$$

This simplified evaluation corresponds to an empirical single-function capacity that can be regarded as a reduced form of the total capacity in IPC theory, which reconstructs multiple orthogonal basis functions of the input.

Despite this simplification, the obtained results as shown in Fig. 15 are consistent with the predictions of IPC theory. For low traffic densities, *C* is small because vehicle interactions are weak and the degree of nonlinearity is small. As the density increases, *C* grows and reaches its maximum in the medium-density regime ($\bar{N}_c \approx 7$–9), but decreases on the high-density side, where excessive interactions cause response saturation and, in some trials, negative values of capacity. These negative values suggest that the system’s sensitivity to input variations is diminished under congested conditions. Therefore, the medium-density regime exhibits the highest information-processing capacity, achieving an optimal balance between linear memory and nonlinear computation. This regime coincides with the density range where RTRC attains the best computational performance.

Finally, while the algorithmic computational complexity on a digital computer can be quantitatively estimated, the intrinsic complexity of the observed physical phenomena has not yet been fully quantified. In HRC, the observed dynamics themselves are interpreted as performing a form of computation. This perspective is consistent with the concept of “Computing Nature” ^53^, which regards natural dynamical processes as embodiments of computation. From this viewpoint, HRC exploits not only the algorithmic complexity in the Turing sense but also the structural organization of traffic dynamics as a computational resource. Although the learning process itself is limited to convex optimization through ridge regression, the underlying physical computational complexity–that is, the complexity of the computational process spontaneously emerging from the system’s dynamics–remains to be clarified. Theoretical elucidation of the dynamical conditions under which such naturally observed systems realize sufficient information-processing capacity is an important direction for future research.

## Conclusion

We proposed Harvested Reservoir Computing as a general methodology for exploiting real-world dynamics as reservoirs. For this study, we demonstrated this concept through RTRC, which leverages traffic flow dynamics. Because HRC treats undesigned real-world dynamics as reservoirs, the observed dynamics do not always function as reservoirs effectively. Instead, reservoir properties emerge or vanish depending on the environment and conditions, which is a distinctive feature of harvested reservoir computing.

For this study, we investigated the conditions under which traffic flow can act as an effective harvested reservoir for prediction. Among the parameters influencing traffic dynamics, we specifically examined traffic density, which varies naturally in response to travel demand. To identify the appropriate density range for RTRC, we evaluated computational performance under different densities through prediction tasks using scaled model experiments and function approximation in numerical simulations. Results from both experiments indicated the intermediate density region as the region in which traffic flow dynamics performed as a high-accuracy reservoir for computing. Furthermore, analysis of the simulation results suggests that this optimal density corresponds to traffic conditions near the critical point of congestion onset, where correlation is visible between memory capacity and road capacity. These results suggest the possibility of estimating optimal traffic conditions for RTRC, even for real-world traffic, where observational data indicate that density changes dynamically.

Our findings suggest the potential of HRC-based approaches for practical applications such as real-time traffic forecasting, adaptive traffic light control, and data-driven reuse of existing traffic measurements. They indicate that new forms of computation can be realized without requiring additional infrastructure or sensor deployment.

A recent study has also advanced, independently, related perspectives on road traffic as a reservoir for computing. Chu et al. demonstrated numerically that shock waves induced by heterogeneous driving policies can enhance machine learning performance in a vehicular reservoir computing framework^72^. By contrast to that demonstration, our study, based on experimentation, examines traffic density and its relation to congestion onset. These differences arise from distinct model settings and assumptions, but both illustrate the growing attention to traffic dynamics as computational resources. This growing body of research can be expected to contribute to further development of HRC and stimulation of future directions for both theoretical refinement and practical applications.

In a broader context, recent advances in unconventional computing indicate that diverse real-world dynamical systems–beyond traffic–are increasingly recognized as potential computational resources. In mechanical systems, the emerging field of mechano-intelligence exploits intrinsic physical properties such as elasticity, vibration, and structural dynamics as media for information transformation^73,74^. This approach integrates control and computation by leveraging mechanical flexibility and nonlinearity instead of relying on complex sensors or controllers, realizing embodied intelligence. Such developments suggest a paradigm shift in AI design–from software-centric architectures toward embodied cognition, where the physical dynamics of hardware and body act as active computational resources. Meanwhile, biological reservoirs exhibit outstanding advantages in both energy efficiency and plasticity over other PRC domains. Wetware computing, which utilizes living neurons or organic materials as computational substrates, offers a far more energy-efficient alternative to silicon-based computation^26^. Biological neurons can process information with orders-of-magnitude lower energy consumption than digital processors, and bio-based reservoirs also promise a sustainable, low-impact form of green computing. These cross-disciplinary developments form a growing movement that reinterprets physical and biological processes of the real world from an information-processing perspective. Harvested Reservoir Computing (HRC), as part of this broader paradigm of unconventional computation, is expected to further accelerate and evolve within this emerging landscape.

## Supplementary Information

Supplementary Information.

## Data Availability

The datasets and codes used and analyzed for the current study are available from the following link: https://github.com/fuku-zaki/HRCRTRC.git.
