# Supplementary material for: Harvested reservoir computing from road traffic dynamics
Source: Sci Rep. 2025 Nov 27;15:45547. doi: 10.1038/s41598-025-30016-2 (PMC12749081; doi:10.1038/s41598-025-30016-2)
Supplement: Supplementary file 1 — Supplementary Information. [file 41598_2025_30016_MOESM1_ESM.pdf]

Supplementary Information
for
Harvested reservoir computing from road traffic dynamics

Ryunosuke Fukuzaki[1,3], Takahiro Noguchi[2], and Hiroyasu Ando[3,*]

[1]Graduate School of Science and Technology, University of Tsukuba, Ibaraki, 305-8573, Japan
[2]Faculty of Economics, Keio University, Tokyo, 108-0073, Japan
[3]WPI-Advanced Institute for Materials Research (WPI-AIMR), Tohoku University, Sendai, Miyagi, 980-8577, Japan
[*]Corresponding author: hiroyasu.ando.d1@tohoku.ac.jp

A Scaled Traffic System

A.1 Scaled Autonomous Vehicle

The vehicles used are based on commercially available RC cars, specifically the mini-Z (Kyosho Corp.). Each vehicle is 180 mm long and 69 mm wide, representing a 1/27 scale of an actual car. Each vehicle is equipped with ToF sensors, Hall sensors, IrDA, and Felica. Each is designed to follow lines while avoiding collisions with vehicles ahead. The maximum speed of the vehicle is 617 mm/s, which corresponds to 60 km/h when scaled up to real-world size. When traveling at maximum speed, the vehicle can operate continuously for 2 hr and 7 min.

Each vehicle is equipped with three ToF sensors on the front: one sensor is placed in the center; the other two are angled at 30 deg to the left and right. The vehicles calculate the distance between themselves and obstacles, including other vehicles, and adjust their speed accordingly by accelerating, decelerating, or stopping. The acceleration is constant, but its deceleration changes in two patterns depending on the distance to the obstacle ahead. If the distance to the obstacle is between 140 mm and 600 mm, then the vehicle calculates the relative speed to the obstacle and controls its speed to maintain that distance. If the distance is less than 140 mm, then the motor applies the brakes. These driving rules are applied uniformly to all vehicles.

In addition, each vehicle is equipped with seven Hall sensors on its front. The vehicles, which recognize magnetic markers embedded in the course as route markers using the onboard Hall sensors, follow these markers while driving. Additional markers (intersection markers) are embedded before intersections, enabling them to recognize the upcoming intersection. When the vehicle detects an intersection marker, it is programmed to either go straight or to make a right or left turn according to a pre-set steering angle. They have no specific destination. They continue driving according to the rules presented above.

A.2 Traffic Light Setup

The two traffic lights on the course are configured with identical settings. LEDs are installed at three locations: 500 mm before the traffic light, 250 mm before the traffic light, and at the stop line. Vehicles detect infrared signals from the LED lights, which enables them to recognize the traffic signal and to ascertain whether they can proceed straight.

If the vehicle recognizes a green light at the stop line, then it will start moving unless it is already decelerating. Otherwise, it accelerates to the target speed. If the vehicle recognizes a yellow or red light, then it stops at the designated stop line.

B Grid Traffic Simulation

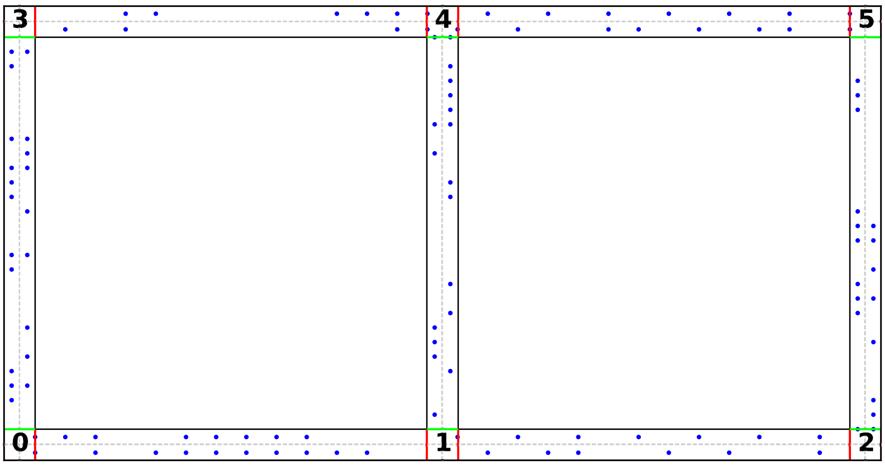

Supplementary Figure B1: Grid road network used in traffic simulations. The layout consists of 2 × 3 intersections with bidirectional roads. Vehicles follow traffic signals and obey collision-avoidance rules.

B.1 Traffic Simulation Setup and Data Processing

In grid traffic simulation, vehicles travel along the course following the optimal velocity model. The optimal velocity model is a representative one-dimensional car-following model that determines vehicle motion based on the optimal velocity V_{opt} corresponding to the headway distance h from the preceding vehicle. All vehicles obtain their optimal velocity from the optimal velocity function $V_{\mathrm{opt}}(h) = \tanh(h-3)+\tanh(-2)$. In addition, a minimum headway distance of $h_{\mathrm{min}} = 1$ is introduced to prevent unrealistic situations in which vehicles are positioned extremely close to one another. Figure B1 portrays a snapshot of the road network and the vehicles running on it (marked in blue). In addition, a snapshot of vehicle movements on the effectively 2×3 network used in the link-length heterogeneity experiment is also presented to illustrate the traffic behavior under heterogeneous road conditions, as shown in Fig. B2. An example of the measured data

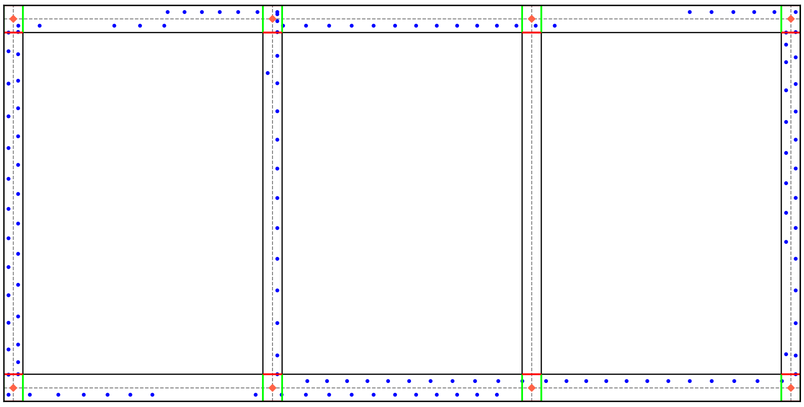

Supplementary Figure B2: Snapshot of vehicle movements on the effectively 2×3 network used in the link-length heterogeneity experiment. One of the vertical roads was closed to traffic, resulting in a nonuniform topology with heterogeneous road lengths.

is presented in Fig. B3. Furthermore, Fig. B4 depicts the results of the transformation applied to the measurement data in Fig. B3. Specifically, m_r and m_i were smoothed and normalized respectively, for each road and intersection, and are presented as $\tilde{m}_r$ and $\tilde{m}_i$. From these, the transformed values $g(\tilde{m}_r)$ and $g(\tilde{m}_i)$ were obtained, where $g(\tilde{m}) = \tanh\big(3(\tilde{m} - 0.5)\big)$. This function provides a gentle nonlinear transformation: $\tilde{m}$ is mapped into the nearly linear region of the tanh function (range $[-1.5, 1.5]$).

B.2 Memory Capacity (MC)

The delay task for measuring memory capacity (MC) assesses how well the RC model can retrieve past inputs and reproduce them as current outputs. In other words, using the reservoir $x(k)$ driven by the input, the model estimates the previous input sequence $s(k-\tau)$ delayed by τ steps, thereby reconstructing the earlier inputs. The value MC_τ, defined in Eq. (B1), represents the degree of agreement between the delayed input sequence $s(k-\tau)$ and the reconstructed sequence $h_\tau(k)$, which is learned to reproduce $s(k-\tau)$. Because this is equivalent to calculating the coefficient of determination, the possible range of MC_τ is $[0, 1]$. If $s(k-\tau)$ is reconstructed accurately as the output of $r(k)$, then MC_τ takes a value close to 1.

$$\mathrm{MC}_\tau = \frac{cov^2(s(k-\tau)), h_\tau(k)}{var(s(k-\tau)var(h_\tau(k)))} \tag{B1}$$

Then, MC is defined as the cumulative sum of the delay-specific memory contributions MC_τ, as presented below.

$$\mathrm{MC} = \sum_{\tau=1}^{\tau_{max}} \mathrm{MC}_\tau \tag{B2}$$

B.3 Finite-sample Variance of MC

To quantify the finite-sample variance of MC, a circular block bootstrap ($B = 100$, block length $= 22$) was applied to a representative seed under the same condition as the delay task ($\tau_{\max} = 2000$, sequence length $= 4000$). The block length was determined as the lag

at which the autocorrelation of the input sequence decayed below $1/e$. For the traffic-flow time series used in this study, this lag was approximately 22 steps, corresponding to the temporal correlation scale of local vehicle interactions. Table B1 summarizes the mean, 95% confidence intervals, and variances of the total MC for each $\bar{N}_c$. The confidence intervals were narrow, with a relative width of ±4–5%, and the relative standard deviation was approximately 2–3% across densities, indicating that the finite-sample effect is minor and the estimated MC is statistically reliable.

Note that the bootstrap estimates tend to be slightly smaller than those in Fig. 8b of the main text because the block resampling weakens long-range temporal correlations at the block boundaries, leading to a conservative estimate of MC without affecting the overall trend.

Supplementary Table B1: Bootstrap estimates of total MC (mean, 95% confidence interval, and variance) across densities.

$\bar{N}_c$	Mean	95% CI (low)	95% CI (high)	Variance
1	658.1	626.4	688.1	284.5
2	675.6	640.4	706.5	275.2
3	680.7	648.9	715.6	272.1
4	684.5	655.3	713.9	256.5
5	694.6	659.3	731.4	309.6
6	700.1	669.8	738.3	296.6
7	708.7	678.6	745.6	281.0
8	708.3	678.2	740.4	251.5
9	715.5	679.9	751.1	311.5
10	715.7	681.5	758.7	322.6
11	717.6	687.2	757.1	286.6
12	715.9	684.0	755.3	314.5
13	722.0	683.3	760.4	340.3
14	718.0	687.2	751.1	271.9

B.4 Input Signal and Approximation Task

To ensure that MC_τ is independent of the input signal, $s(k)$ is set as a random sequence. In the traffic simulation, the input consists of the traffic light patterns at all intersections. Because traffic lights have two states (red and green), $s(k)$ is a binary random sequence. All traffic lights are synchronized, meaning that when one direction of an intersection has a green light, the perpendicular direction has a red light. Because traffic simulation uses the volume of traffic passing through intersections m_i as the reservoir, the system exhibits a response that is highly dependent on the input sequence. Therefore, in this delay task, the goal is to reconstruct the past states of $f(s(k))$, where $f(s(k))$ is obtained by application of amplitude attenuation based on the previous signal duration to a sine function. Figure B5 presents $s(k)$ and $f(s(k))$.

First, the traffic light display pattern is given as

$$s_i = \begin{cases} 1 & (x_i < 5) \\ 0 & (x_i \geq 5), \end{cases}$$

where x_i is an ith integer chosen randomly from a uniform distribution within the range $0 \leq x_i < 10$. This pattern determines the green or red state of the signal, where 1 corresponds to the green light, and 0 corresponds to the red light.

Next, in the duration setting, each signal value maintains a constant value for a randomly determined duration τ_i.

$$\tau_i = 10 \times y_i$$

In that equation, y_i is an integer randomly chosen within the range $5 \leq y_i \leq 10$. The signal value s_i is repeated over the duration τ_i.

$$s(k) = s_i, \quad t \in \left[\sum_{j=0}^{i-1} \tau_j, \sum_{j=0}^{i} \tau_j \right).$$

The traffic light display $s(k)$, which is generated as a spike signal, is transformed into a damped sinusoidal wave $f(s(k))$. The sinusoidal function $I(t)$ is given as

$$I(k) = A \sin \left(\frac{10k}{\pi T_\tau} \right),$$

where $T_\tau = 50$ is the fixed decay duration, equal to the minimum duration of the traffic light. The decay function $D(t)$ is represented as shown below.

$$D(k) = \exp \left(1 - \frac{2\pi k}{T_\tau} \right)$$

The sign of $f(s(k))$ changes depending on the value of the step signal. Specifically, the amplitude is $A = 1$ for a green light, and $A = -1$ for a red light. Combining the decay function and the sinusoidal wave, $f(s(k))$ is expressed as presented below.

$$f(s(k)) = A \cdot D(k) \cdot I(k) \quad \begin{cases} A = 1 & (s_i = 1) \\ A = -1 & (s_i = 0) \end{cases}$$

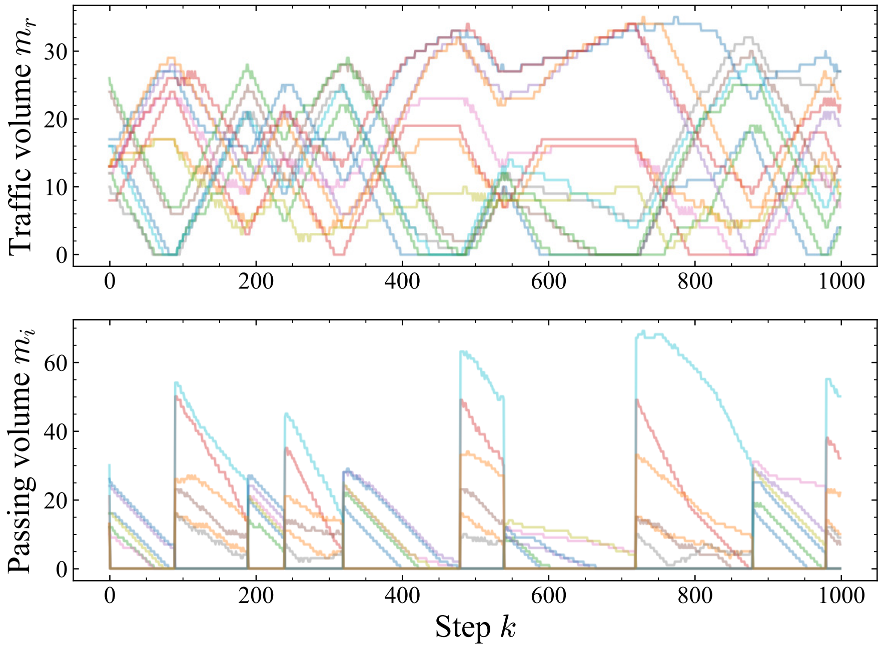

Supplementary Figure B3: Examples of traffic state measurements in the grid road simulation, showing (top) the number of vehicles per road and (bottom) the passing volume at intersections over time.

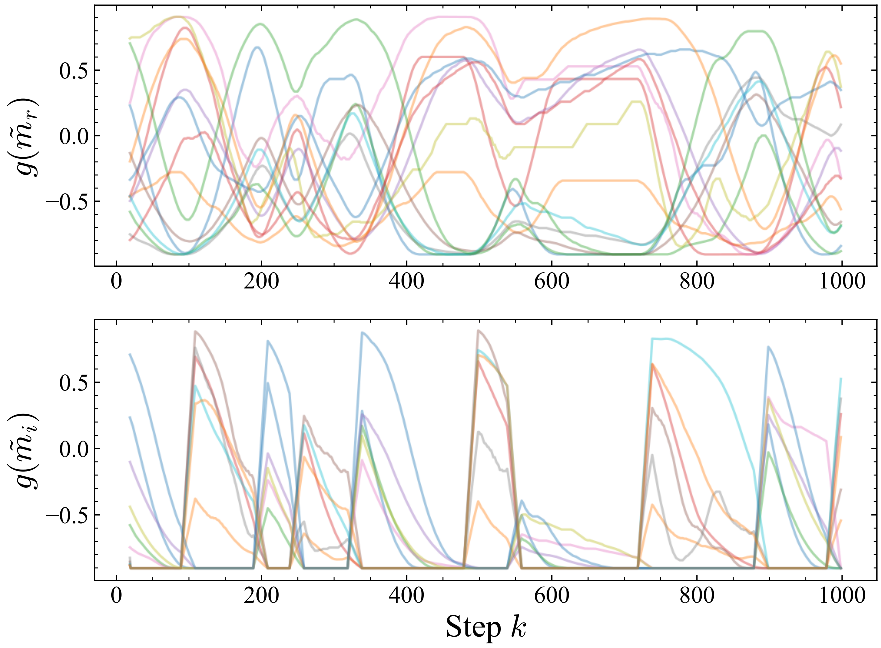

Supplementary Figure B4: Transformed traffic measurements used as reservoir inputs. Raw measurements are normalized and passed through a smooth nonlinear transformation to enhance dynamic expressiveness.

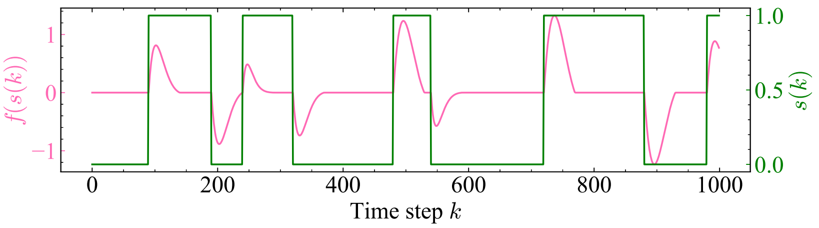

Supplementary Figure B5: Traffic light display input sequence $s(k)$ (green) and its nonlinearly transformed sequence $f(s(k))$ (pink).

C Detailed Analysis

C.1 Sensitivity Analysis for Ridge Coefficient

We performed a sensitivity analysis of the ridge coefficient $\beta \in \{0.1, 1, 10, 100, 1000\}$. While the optimal β varied slightly across tasks, the density–accuracy curve (Fig. 7 of the main text) was qualitatively unchanged; the medium-density optimum persisted for both targets.

Supplementary Table C1: Sensitivity of NRMSE to the regularization coefficient β for two prediction tasks in the scaled traffic experiment ($V_{\max} = 300$ mm/s).

	(a) Power consumption prediction					(b) Average speed prediction				
**Num**	**0.1**	**1**	**10**	**100**	**1000**	**0.1**	**1**	**10**	**100**	**1000**
4	1.0150	0.8564	0.8235	0.8572	0.8688	1.2030	1.1443	1.0650	1.0280	1.0048
6	0.4781	0.4510	0.4333	0.4513	0.4540	0.7202	0.7100	0.6923	0.6955	0.7040
8	0.5872	0.5602	0.5317	0.5384	0.5417	0.7297	0.6980	0.6604	0.6577	0.6582
10	0.7570	0.7242	0.7085	0.6701	0.6007	0.9117	0.8703	0.8999	0.8814	0.7191

C.2 Stability Analysis for Observed Dynamics

Recorded states $x(k)$ were standardized per coordinate and reduced by principal component analysis (PCA) to the smallest dimension d explaining 95% cumulative variance (typical $d = 15$–37).

On the compressed sequence $\tilde{x}(k) \in \mathbb{R}^d$, the Rosenstein–Kantz nearest-neighbor method was applied with a Theiler window $T_{\text{th}} = 8$ and $k_{\text{NN}} = 5$. The slope of $\langle \log d(\Delta t) \rangle$ over the initial linear region $\Delta t \in [2, 15]$ provided the LLE (per step). Uncertainty was evaluated as a bootstrap standard error over 200 resamples.

Local linear models $x_{k+1} \approx A_k x_k$ were fitted in sliding windows (length 50, step 10) with ridge regularization ($\beta = 10^{-3}$). The mean logarithmic growth rate

$$\lambda_{\text{avg}} = \frac{1}{T} \sum_k \log \sigma_{\max}(A_k)$$

was calculated from the maximal singular value $\sigma_{\max}(A_k)$ of each local propagator.

Table C2 lists the LLE, standard error (SE), fit range, PCA dimension, and λ_{avg} for all $(N_c, V_{\max})$ conditions. The estimated LLE values are all positive but remain small, approximately in the range of 0.006–0.016 per step, indicating weak local divergence

rather than chaotic instability. Both LLE and λ_{avg} decrease as the number of vehicles N_c increases, suggesting that the observed dynamics become more reproducible at higher densities. In contrast, variation with the maximum speed $V_{\max}$ is minor, consistent with the result in Fig. 7 of the main text showing that prediction accuracy is largely insensitive to $V_{\max}$.

Supplementary Table C2: Largest Lyapunov exponent (LLE), bootstrap standard error (SE), and mean local growth rate λ_{avg} estimated from the scaled traffic experiment. The fit range is $\Delta t \in [2, 15]$ for all conditions.

N_c	$V_{\max}$ (mm/s)	PCA comps d	LLE	SE	λ_{avg}
4	300	16	0.0136	0.00139	4.09
4	450	15	0.0161	0.00152	3.53
4	600	18	0.0092	0.00122	3.45
6	300	21	0.0078	0.00094	2.70
6	450	19	0.0145	0.00155	2.67
6	600	22	0.0117	0.00142	2.71
8	300	27	0.0085	0.00119	2.18
8	450	28	0.0061	0.00096	2.09
8	600	31	0.0076	0.00076	2.01
10	300	32	0.0062	0.00094	1.85
10	450	36	0.0066	0.00099	1.86
10	600	37	0.0076	0.00137	1.93

C.3 Real-Time Evaluation and Benchmark Model Configurations

This appendix presents the procedure and results of the real-time evaluation conducted under the same condition as the main benchmark experiments. All models were executed under identical computational conditions. For each trained model, single-step inference was repeated 1000 times to estimate the mean per-step inference latency L_{step} and its standard deviation (jitter) J. Given the physical update period $\Delta t^* = 5$ s, we define

$$\mathrm{RTF} = \frac{\Delta t^*}{L_{\text{step}}}.$$

For RTRC, we observed $L_{\text{step}} \approx 2.2\ \mu$s and $T_{\text{train}} \approx 0.15$ ms, corresponding to RTF $\approx 2.3 \times 10^6$. Under the maximum configuration ($N_c = 10$), additional measurements yielded 1.6–1.7 μs for inference and approximately 0.9 ms for training, consistent with the results at $N_c = 6$. These findings agree with the theoretical scaling of $O(N_x)$ for inference and $O(N_x^2)$ for ridge regression training, indicating that RTRC maintains real-time performance as the state dimension increases.

Applying the same protocol to the baselines, ESN, LSTM, and ARIMAX exhibited per-step inference latencies of approximately 25–50 μs, 70 μs, and 1.0–1.2 ms, respectively, with training times ranging from several milliseconds to several seconds. Hence, RTRC is one to four orders of magnitude faster in both training and inference while attaining competitive or superior accuracy in the main tasks, making real-time operation and online adaptation feasible.

Hyperparameter search ranges. For RTRC, the ridge coefficient $\beta \in \{0.1, 1, 10, 100, 1000\}$ was selected based on validation NRMSE. For ESN, a 20-trial random search was conducted over reservoir size {200, 300, 400, 500}, spectral radius [0.6, 1.1], input scaling [0.1, 1.0], leak rate [0.1, 1.0], and ridge $\beta \in$ {0.1, 1, 10, 100, 1000}, selecting the best by validation NRMSE. The LSTM used one layer with 32 hidden units (window length 5), the Adam optimizer with learning rate 10^{-3}, 80 epochs, and batch size 64; the best configuration within this predefined setup was selected by validation NRMSE. ARIMAX used $(p, d, q) = (2, 0, 2)$ as the base configuration, automatically switching solvers or orders when convergence failed. All settings were applied to the same preprocessed inputs and targets to ensure a consistent and fair comparison.

Computational complexity. For RTRC, inference requires a single matrix–vector multiplication, yielding $O(N_x)$ complexity with respect to the state dimension N_x, and ridge regression training is $O(N_x^2)$ for solving the normal equations. ESN and LSTM involve recurrent state updates or backpropagation through time, which typically scale as $O(TN_x^2)$ and $O(TN_h^2)$ per sequence, respectively, where T is the sequence length and N_h the number of hidden units. ARIMA repeatedly optimizes likelihood functions, leading to $O(Tp^2)$ per iteration, where p is the autoregressive order. These relationships align with the measured results and explain why RTRC retains real-time performance as the system size increases.

D Experimental and Simulation Parameters

All quantitative parameters used in the scaled physical experiments and the grid traffic simulations are summarized in Table D1. These values ensure the reproducibility of the results presented in the main text. All parameter values correspond to those used in the main experiments and enable full reproducibility of the results. Additionally, the inter-vehicle variation in measured speed at the same marker position during free-run tests was evaluated to quantify sensor consistency and noise level. The systematic bias across vehicles was found to be within approximately 5% at maximum.

Supplementary Table D1: Experimental and simulation parameters for reproducibility.

Category	Parameter / Symbol	Value / Description
**Scaled model experiment**		
Sampling frequency	f_s	1 Hz (data logger rate)
Time-multiplex interval	τ	5 s (step interval for Eq. (4) of the main text)
Number of lags	N_l	5 (total horizon $H = \tau N_l = 25$ s)
Number of vehicles	N_c	4–10 (density levels in Fig. 7 of the main text)
Number of variables	N_d	3 (speed, acceleration, power)
Ridge coefficient	β	10 (fixed; sensitivity in Table C1)
Data length (train/test)	—	200 / 100 time points per density and task
Tasks	—	(I) Total power, (II) Average speed
**Grid traffic simulation (default)**		
Network topology	—	2×3 intersections (14 links; Fig. 5 of the main text and Fig. B1)
Road length	L	50
Number of roads	N_r	14
Number of intersections	N_i	6
Average number of vehicles	$\bar{N}_c$	1–15 (average density per road)
Total number of vehicles	N_c	14–210 ($N_c = \bar{N}_c N_r$)
Number of lags	N_l	20
Vehicle model	—	Optimal velocity model
Ridge coefficient	β	10 (fixed)
Sequence length for MC	—	4000 steps ($\tau_{\max} = 2000$)
**Evaluation metrics**		
Normalized RMSE	—	NRMSE = RMSE/σ_{target}
Root mean square error	—	RMSE = $\sqrt{\sum_t (y_t - \hat{y}_t)^2/N}$
Memory capacity	MC	Defined by Eqs. (B1)-(B2) in Section B
